# Using the national electronic prescription system to determine the primary non-adherence to medication in the Czech Republic

**DOI:** 10.3389/fphar.2023.1128457

**Published:** 2023-03-22

**Authors:** Jan Bruthans, Jiří Berger, Ján Šoltés, Pavel Michálek

**Affiliations:** ^1^ Department of Anesthesiology and Intensive Care, General Teaching Hospital, Prague, Czechia; ^2^ Department of Biomedical Technology, Faculty of Biomedical Engineering, Czech Technical University, Prague, Czechia; ^3^ Institute of Pathological Physiology, First Faculty of Medicine, Charles University, Prague, Czechia; ^4^ Department of Anaesthesia, Antrim Area Hospital, Antrim, United Kingdom

**Keywords:** medication adherence, primary medication non-adherence, electronic prescribing, filling prescription, eHealth

## Abstract

The primary medication non-adherence occurs when a patient does not collect his or her newly prescribed medication. Various studies give estimates that this occurs between 0.2 percent and 74 percent. Recently, this topic has been researched by analyzing data in national electronic prescription systems. The database of the Czech electronic prescription system was used to obtain the number of all prescriptions issued and collected in 2021 for fifty particular substances (associated with six medication groups). Additionally, a similar query was performed with an additional criterion that the same substance had not been prescribed within the last 365 days. The data were obtained separately in five age categories. The total number of prescriptions analyzed in this study was over 21 million, which represents almost 30 percent of all prescriptions issued in the Czech Republic in 2021. The primary medication non-adherence in the selected substances was 4.56 percent, which negatively correlates (rxy = 0.707) with the age of a patient. There is a higher primary non-adherence in the Psychoanaleptics and Lipid modifying medication groups than in the whole studied sample (*p* < 0.05). Lipid-modifying medication group and several other particular substances showed a larger difference between primary non-adherence and overall non-adherence, indicating issues in the initiation of these drugs. The results of our study are following earlier studies with similar methodologies from other countries. However, the difference between primary non-adherence and overall non-adherence had not been observed in other studies before. The electronic prescription system proved to be a valuable tool for conducting this type of research.

## 1 Introduction

In his time, Hippocrates made an interesting observation that “some patients do not take prescribed medicine and many of them later complain that treatment does not work” (Brown and Bussell, 2011). The medication adherence to the treatment comprises these steps—1) initiation, that is, taking the first dose; 2) implementation, that is, taking further doses according to the prescribed schedule and 3) discontinuation, that is, terminating the treatment at the right moment (Vrijens et al., 2012). Generally, medication adherence is determined from the perspective of not collecting a prescription, therefore being described as medication non-adherence (MN), which is further divided into primary medication non-adherence (PMN) and secondary medication non-adherence (SMN) (Raebel et al., 2013). Thus, PMN is an integral part of an initiation, whereas SMN relates more to implementation.

The widely-used definition states that “PMN occurs when a new medication is prescribed for a patient, but the patient does not obtain the medication or an appropriate alternative within an acceptable period of time after it was prescribed” (Adams and Stolpe, 2016). Yet, this definition is not very accurate as there is no specific criterion of what is “a new medication”, and there is no unequivocal time interval when the prescription is seen as not collected and therefore “medication is not obtained.” Occasionally, the term initial medication non-adherence is used instead of PMN (Aznar-Lou et al., 2017). If the first prescription was claimed, but the patient failed to do so in some subsequent prescription, the term SMN is used. SMN has been studied for a long time using pharmacy records and questioning patients who have already started their treatment. SMN has also been employed as a target for quality improvement in medicine.

The research on PMN was difficult in the past, as no simple way to identify these cases existed, patient questionnaires (da Costa et al., 2015; Wamala et al., 2007) were employed- or insurance funds records were analyzed (Harsha et al., 2019). This has changed as national outpatient electronic prescription systems (OEPS) have gradually spread in many developed countries, leading to successful PMN studies in Denmark (Hempenius et al., 2022), Estonia (Laius et al., 2018), Poland (Kardas et al., 2020), Spain (Rodriguez-Bernal et al., 2018), Canada (Singer et al., 2022), and the United States (Fischer et al., 2010). Various PMN studies focused on different medications, but nowadays even complex studies analyzing multiple medication groups exist (Kardas et al., 2019). Moreover, the results of PMN at present are very disparate, as PMN is reported between 0.2 and a staggering 74.0 percent (Cheen et al., 2019).

Despite having an advanced and functional national OEPS (Bruthans, 2019) no PMN study has been conducted in the Czech Republic until now. Also, according to our knowledge, the data in the Czech OEPS have not been used in any kind of pharmacology study so far. Therefore, the aim of this study is to provide a detailed view of primary medication non-adherence in the Czech Republic using the national OEPS and also to show-how—the data in OEPS can be used in such studies in general.

## 2 Methods

The design of this study is retrospective and non-interventional. Data stored in the Czech OEPS system were used. It is the first study of such kind in the Czech Republic. As the basic definition of MN implies, two data sets were required—the number of prescriptions issued, and the number of prescriptions collected.

To clarify the uncertainties of PMN definition we defined “new medication” as any substance with the same Anatomical Therapeutic Chemical (ATC) code (e.g., regardless of the strength of the medication or manufacturer) not prescribed in the previous 365 days. We set “not obtaining the medication” as not collecting a prescription until the expiration of the prescription or in 60 days, whichever comes first. Usually, the former variant is to be expected as the Czech prescriptions are (if not stated by the physician otherwise) valid for 15 days. We have chosen the year 2021 to be analyzed as it is the last year for which the whole data set was available at the time of drafting this article.

Six different groups of medication were opted for to cover a broad spectrum—Antidiabetics, Antithrombotic agents, Medication for the cardiovascular system, Lipid modifying agents, Anti-infectives, and Psychoanaleptics. After consultation with specialists (internal medicine specialist for the first four groups, microbiologist for the fifth, and psychiatrist for the last one) the most relevant ATC substances were included—in total 50 of them. The complete list and its grouping into subsets are depicted in Table 1. No subgrouping was designated in the anti-infectives category.

**TABLE 1:** ATC substances chosen for PMN analysis.

Group	Subgroup	ATC substance (name)	ATC substance (code)
Antidiabetics medication	Biguanides	metformin	A10BA02
	Sulfonylureas	gliclazide	A10BB09
		glimepiride	A10BB12
	α-Glucosidase inhibitors	Acarbose	A10BF01
	Dipeptidyl peptidase 4 inhibitors	sitagliptin	A10BH01
	SGLT2 Inhibitors	dapagliflozin	A10BK01
		repaglinide	A10BX02
Antithrombotic agents	Vitamin K antagonists (VKAs)	warfarin	B01AA03
	Platelet aggregation inhibitors	clopidogrel	B01AC04
	Novel oral anticoagulants	dabigatran	B01AE07
		rivaroxaban	B01AF01
Medication for the cardiovascular system	Diuretics	hydrochlorothiazide	C03AA03
		indapamide	C03BA11
		furosemide	C03CA01
		spironolactone	C03DA01
		eplerenone	C03DA04
	b-blockers	metoprolol	C07AB02
		bisoprolol	C07AB07
		nebivolol	C07AB12
		carvedilol	C07AG02
	ca-channel blockers	amlodipine	C08CA01
		lacidipine	C08CA09
		diltiazem	C08DB01
	ACE-inhibitors	enalapril	C09AA02
		perindopril	C09AA04
		ramipril	C09AA05
		quinapril	C09AA06
	Angiotensin II-receptor blockers	losartan	C09CA01
		valsartan	C09CA03
Lipid modifying agents	HMG CoA reductase inhibitors	simvastatin	C10AA01
		atorvastatin	C10AA05
		rosuvastatin	C10AA07
	Fibrates	fenofibrate	C10AB05
Anti-infectives	*	doxycycline	J01AA02
		amoxicillin	J01CA04
		amoxicillin and β-lactamase inhibitor	J01CR02
		cefuroxime	J01DC02
		cotrimoxazole	J01EE01
		roxithromycin	J01FA06
		clarithromycin	J01FA09
		azithromycin	J01FA10
		clindamycin	J01FF01
		ciprofloxacin	J01MA02
		furantoin	J01XE01
Psychoanaleptics	Selective inhibitors	paroxetine	N06AB05
		sertraline	N06AB06
		escitalopram	N06AB10
	Other antidepresives	mirtazapin	N06AX11
		venlafaxine	N06AX16
		vortioxetine	N06AX26

*No subgroup set for the Anti-infectives group.

The Czech Republic OEPS (named eRecept) does not differ significantly from other OEPS in most EU countries (Bruthans, 2020). At present more than 98 percent of prescriptions in the Czech Republic are issued electronically (over 72 million per year in 2021 compared to over 1.5 million per year paper-based in 2021). The State Institute for Drug Control (SIDC) is responsible for running the eRecept system. OEPS consists of a central repository where every issued prescription is stored, along with data of an issuing physician, and whether, where, and to which extent the prescription was collected. Also, the data about the patient are stored, and by a connection to the Czech citizen registry using name and date of birth, the OEPS matches every issued prescription to a particular patient. By using this feature the prescriptions can be collected not only by using the unique identifier of a prescription but the pharmacy can also dispense the prescribed medication whenever an ID/passport is presented. Regrettably, ATC codes are not used in the OEPS database, unique drug SIDC identifier of every drug registered in the Czech Republic is used instead. This means that the same ATC substance from two manufacturers and/or with different strengths and/or with a different number of pills always gets a different SIDC identifier. So, for example, the information that metformin (A10B02) was prescribed is listed under any of its 855 SIDC identifiers. Similarly, there are 267 identifiers for simvastatin (C10AA01), *etc.*

The Institutional Review Board of the First medical faculty of Charles University and the General Teaching Hospital in Prague was consulted and decided that our study does not require its approval due to the characteristics of this study.

SIDC was asked to provide the following: 1) The number of all electronically issued prescriptions and the number of all collected prescriptions for the whole year 2021, divided into five age groups (under 18 years, 18–39, 39–64, 64–75, 75 and older). 2) The number of prescriptions issued and collected, again divided into the same age groups for every ATC substance which had been chosen for the research (we provided the SIDC with the lists of SIDC identifiers to ensure all possible variants were covered). 3) The number of prescriptions issued and collected as in the previous query, but also meeting the criterion that the same ATC substance was not prescribed within the last 365 days. The inquiry was performed throughout the whole OEPS database from 2021, so every prescription issued electronically that year was analyzed.

Our data request was accepted by the SIDC, however, as the OEPS database is administered by an external provider, the fee of 45.375 Czech Crowns was requested (circa 1815 EUR) for the preparation of the dataset. The fee was covered by the research funds of the General Teaching Hospital and the data were thereafter delivered in the form of a spreadsheet file.

For fundamental statistical analysis (means, calculation of percentage, *etc.*), descriptive statistical methods from MS Excel were used, and subsequently, the parametric type of data was identified. For a more profound analysis, R Studio was used to apply linear (Pearson’s r) and exponential regression analysis. For the comparison of statistically significant differences among the groups, a 1-sample Z-test in the MiniTab application was used. Results with *p* < 0,05 were considered statistically significant.

## 3 Results

**FIGURE 1:**
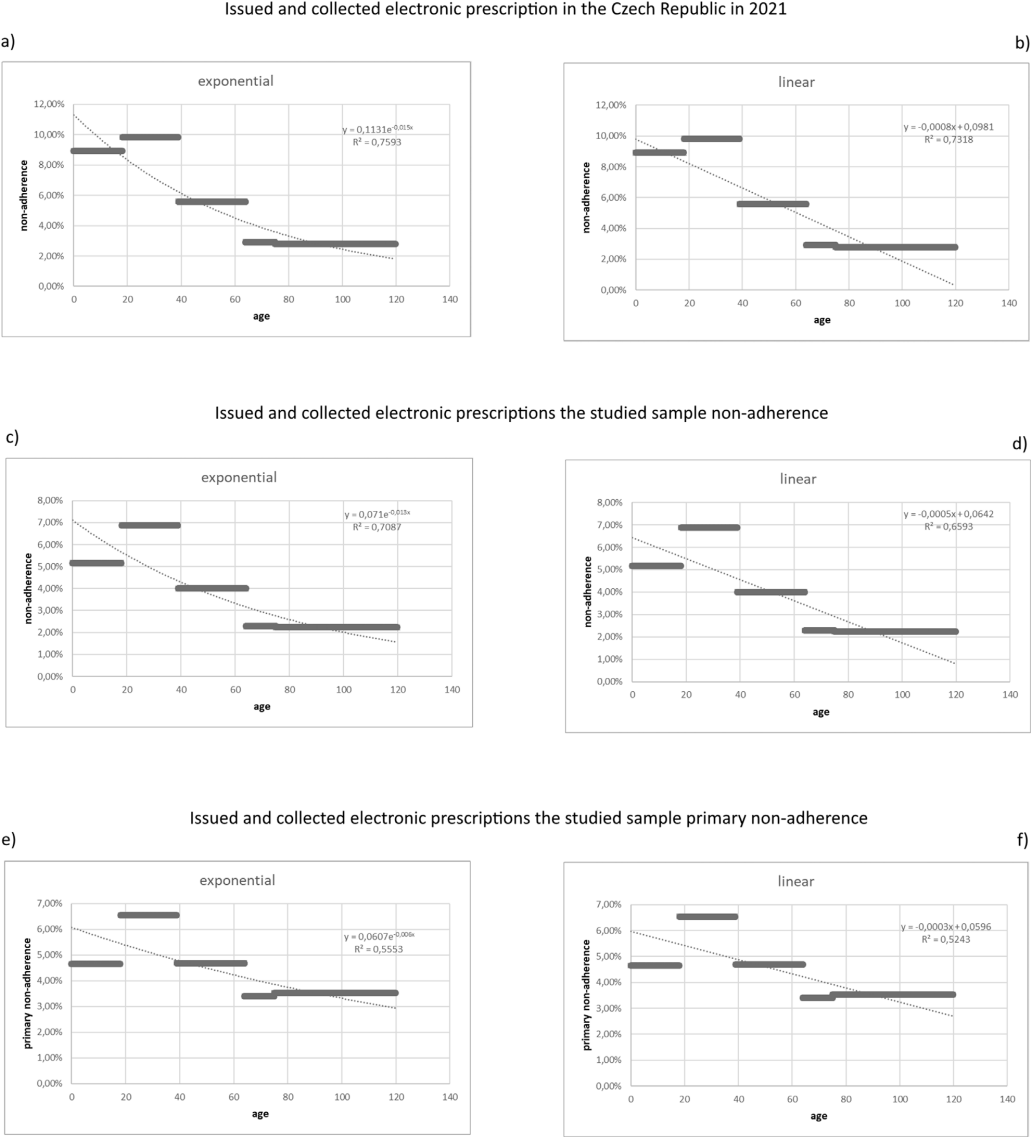
Graphical depiction of the dependences of the non-adherences on the age of the patient.

The total number of all electronically issued prescriptions in 2021 in the Czech Republic was 72,111,659 of which 68,697,607 were collected. This shows the MN amounts to 5 percent, the highest being in the age group 18–39 (9.82 percent), and the lowest in the age group of 75 and older (2.79 percent). All data are set in Table 2. Using linear (Pearson’s r) and exponential regression functions, we interpolated the provided data with a straight line and a curve to evaluate the dependence of the non-adherence on the age of a patient. In both cases, we concluded that there is a dependence of the MN on the age of the patient with a correlation coefficient of 0.883. This is a so-called negative correlation, where the ratio of uncollected electronic prescriptions decreases with the increasing age group. The exponential regression is depicted in Figure 1A), and the linear one in Figure 1B).

**TABLE 2:** Issued and collected electronic prescriptions in the Czech Republic in 2021.

Age group	Issued prescriptions	Collected prescriptions	Non-adherence (%)
<18 years	4,665,286	4,249,552	8.91
18–39	8,478,548	7,645,724	9.82
39–64	24,754,474	23,372,163	5.58
64–75	18,767,654	18,220,761	2.91
75 and older	15,645,697	15,209,407	2.79
Total	72,311,659	68,697,607	5.00

In total there were 21,063,893 electronically issued prescriptions for medications of our sample of 50 ATC substances of which 20,391,202 were collected, so the MN amounts to 3.19 percent. 4,332,690 fulfill the definition of “new medication” so with 4,135,271, this amounts to a PMN of 4.56 percent. The highest MN and PMN were observed in the age group 18–39, and the lowest in 75 and older. Relevant data are set in Table 3—values for collected prescriptions were omitted for the sake of simplicity.

**TABLE 3:** Issued and collected electronic prescriptions in the studied sample—non-adherence and primary non-adherence in the age groups.

Age group	Prescribed (total)	Non-adherence (%)	Prescribed (newly)	Primary non-adherence (%)
<18 years	511,593	5.16	375,148	4.65
18–39	1,393,031	6.88	752,283	6.54
39–64	6,737,544	4.01	1,639,932	4.68
64–75	6,747,207	2.28	897,911	3.40
75 and older	5,674,518	2.23	667,416	3.52
Total	21,063,893	3.19	4,332,690	4.56

If we compare MN in all prescriptions (5.00 percent) with MN in our sample consisting of selected 50 ATC substances (3.16 percent), it might seem that there is a significant difference. Therefore, we performed a statistical evaluation of both data samples according to age groups. First, the mean (x = 6.00), and standard deviation (sx = 3.28) for MN in all prescriptions were counted, then the mean (x = 4.11), and standard deviation (sx = 1.98) for MN in our sample (only with chosen 50 ATC substances) were calculated. The resulting correlation coefficient rxy = 0.98 means a high level of dependence. Therefore, we can conclude that the MN in our sample is statistically very similar to the MN for all electronic prescriptions in the Czech Republic.

We used linear and exponential regression functions in the age groups both for MN and PMN. In the MN case, we concluded that there is a dependence of the ratio of uncollected electronic prescriptions on the age of the patient with a correlation coefficient of 0.82. In the case of PMN, we concluded that there is a dependence of the ratio of uncollected electronic prescriptions on the age of the patient with a correlation coefficient of 0.70. As shown in the graphs (Figure 1C, D) for MN and Figure 1E, F) for PMN) the dependence would be stronger if the youngest age group (under 18) was not included.

The issued and collected prescriptions in our sample were combined into six basic groups and then split into subgroups—all data is shown in Table 4, again omitting the collected prescriptions numbers to simplify the table.

**TABLE 4:** Issued and collected electronic prescriptions in the studied sample—non-adherence and primary non-adherence in the main groups and subgroups of studied substances.

Group	Prescribed (total)	Non-adherence (%)	Prescribed (newly)	Primary non-adherence (%)
**Antidiabetics medication**	**2,513,791**	**2.88**	**162,418**	**3.63**
Biguanides	1,794,875	2.92	106,988	3.79
Sulfonylureas	559,983	2.74	33,221	3.55
α-Glucosidase inhibitors	7,472	5.88	698	7.74
Dipeptidyl peptidase 4 inhibitors	63,523	2.60	8,363	2.83
SGLT2 Inhibitors	87,938	2.71	13,148	2.78
**Antithrombotic agents**	**1,297,480**	**2.57**	**150,745**	**4.05**
Vitamin K antagonists	528,829	1.96	21,336	3.59
Platelet aggregation inhibitors	304,078	2.73	35,013	4.08
Novel oral anticoagulants	464,573	3.17	94,396	4.13
**Medication for the cardiovascular system**	**9,139,285**	**2.50**	**772,764**	**4.49**
Diuretics	2,066,845	2.60	255,985	3.87
b-blockers	3,302,527	2.50	208,366	4.68
ca-channel blockers	1,038,406	2.31	94,200	4.26
ACE-inhibitors	2,168,857	2.51	188,539	5.12
Angiotensin II-receptor block	552,016	2.47	25,268	5.18
**Lipid modifying agents**	**5,061,812**	**2.94**	**400,379**	**5.71**
HMG CoA reductase inhibitors	4,640,851	2.91	371,908	5.62
Fibrates	420,961	3.21	28,471	6.84
**Anti-infectives**	**3,491,579**	**4.62**	**2,723,051**	**4.45**
**Psychoanaleptics**	**2,106,056**	**5.27**	**230,998**	**5.20**
Selective inhibitors	1,374,880	5.00	152,613	5.32
Other anti depressives	731,176	5.79	78,385	4.97

Within our researched groups of ATC substances, two of them showed a significantly higher MN than the average of our studied sample (3.19 percent). The MN in the Anti-infectives group was 4.62 percent, whereas the Psychoanaleptics group had a non-adherence rate of 5.27 percent. Similarly, when we focused on the PMN, we found that the Psychoanaleptics group had a PMN of 5.20 percent and the Lipid modifying group had a PMN of 5.71 percent, both of which were higher rates than the average of the studied sample 4.57 percent (*p* < 0.05).

Our hypothesis that MN in the Antithrombotic and Medication for the cardiovascular system groups was significantly lower than in the whole studied sample was not confirmed. Similarly, lower values of PMN in the Diabetes and Antithrombotic groups were not significant.

The PMN in several subgroups of our studied sample (Vitamin K antagonists (VKAs), B-blockers, Ca-channel blockers, ACE-inhibitors, Angiotensin II-receptor block, HMG CoA reductase inhibitors, and Fibrates) was approximately twice as high as MN. The same finding occurred in the group of Lipid modifying agents and the same trend (albeit without any statistical significance) was observed in the other three groups of our sample (Antidiabetics, Antithrombotic agents, and Medication for the cardiovascular system).

**FIGURE 2:**
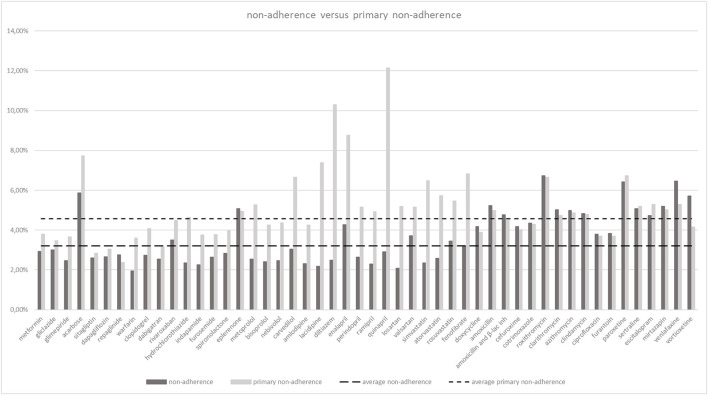
Non-adherence *versus* primary non-adherence by substances.

The number of issued and collected prescriptions for every ATC substance of our research is set in Table 5 and has the same form as the preceding two tables. MN and PMN of every ATC substance are also set in Figure 2.

**TABLE 5:** Issued and collected electronic prescriptions in the selected ATC substances—non-adherence and primary non-adherence.

ATC substance (name)	Prescribed (total)	Non-adherence (%)	Prescribed (newly)	Primary non-adherence (%)
metformin	1,794,875	2.92	106,988	3.79
gliclazide	283,481	3.00	18,445	3.48
glimepiride	276,502	2.47	14,776	3.65
Acarbose	7,472	5.88	698	7.74
sitagliptin	63,523	2.60	8,363	2.83
dapagliflozin	44,369	2.66	7,773	3.05
repaglinide	43,569	2.76	5,375	2.38
warfarin	528,829	1.96	21,336	3.59
clopidogrel	304,078	2.73	35,013	4.08
dabigatran	162,944	2.54	24,751	3.16
rivaroxaban	301,629	3.51	69,645	4.48
hydrochlorothiazide	105,200	2.36	13,786	4.64
indapamide	517,135	2.26	54,279	3.75
furosemide	894,276	2.63	119,333	3.77
sprinolactone	531,500	2.84	66,790	3.96
eplerenone	18,734	5.08	1,797	4.95
metoprolol	1,344,153	2.55	71,523	5.26
bisoprolol	1,392,811	2.41	95,490	4.25
nebivolol	458,283	2.47	36,164	4.37
carvedilol	107,280	3.04	5,189	6.65
amlodipine	1,031,069	2.31	93,996	4.24
lacidipine	10,634	2.17	406	7.39
diltiazem	7,337	2.49	204	10.29
enalapril	29,705	4.28	1,791	8.77
perindopril	1,223,482	2.63	128,424	5.16
ramipril	911,306	2.29	58,184	4.92
quinapril	4,364	2.91	140	12.14
losartan	420,496	2.09	17,974	5.20
valsartan	131,520	3.71	7,294	5.15
simvastatin	159,367	2.35	6,045	6.48
atorvastatin	2,654,986	2.58	198,423	5.73
rosuvastatin	1,826,498	3.44	167,440	5.46
fenofibrate	420,961	3.21	28,471	6.84
doxycycline	214,675	4.18	174,962	3.90
amoxicillin	177,369	5.23	152,986	4.98
amoxicillin and β-lactamase inhibitor	1,000,580	4.78	763,585	4.57
cefuroxime	372,510	4.18	295,602	4.01
cotrimoxazol	313,756	4.35	230,591	4.30
roxithromycin	7,138	6.74	5,842	6.66
klaritromycin	429,351	5.02	35,4205	4.74
azithromycin	385,334	4.99	313,288	4.87
clindamycin	206,863	4.84	156,450	4.80
ciprofloxacin	164,716	3.79	122,843	3.69
furantoin	219,287	3.83	152,697	3.69
paroxetine	92,720	6.42	7,392	6.72
sertraline	591,728	5.07	63,962	5.19
escitalopram	690,432	4.74	81,259	5.30
mirtazapin	332,790	5.20	46,036	5.02
venlafaxine	306,414	6.47	20,794	5.29
vortioxetine	91,972	5.70	11,555	4.15

Several substances exhibited higher non-adherence rates, e.g., diltiazem or quinapril but their frequency of appearance in our sample was inadequately low when compared to other substances. This led to our conclusion that from the statistical point of view, it is not possible to prove any significant difference from the whole sample.

We found three substances (lacidipine, diltiazem, and quinapril) that showed three or four times the PMN when compared to their particular MN. A considerable number of substances in our sample (warfarin, hydrochlorothiazide, metoprolol, carvedilol, amlodipine, enalapril, perindopril, ramipril, losartan, simvastatin, atorvastatin, and fenofibrate) showed approximately twice the PMN when compared to MN.

## 4 Discussion

Compared to several other studies, our MN of 3.19 percent and PMN of 4.56 percent might seem comparatively low. However, this is due to different ways of computing the MN and PMN. Studies with similar complex methodology showed comparable PMN to our study. PMN to antidepressants (based on the pharmacy records and no dispensed antidepressants in the previous 12 months) was observed to be 4 percent (Bauer et al., 2013). PMN to glycemic-lowering, antihypertensive, or lipid-lowering medications (research based on the electronic records, no similar medication prescribed in the previous 24 months, dispensing within 60 days) was noted to be 5 percent (Karter et al., 2009). PMN to oral diabetes prescriptions (defined as no dispensing of a new medication within 60 days of the prescribing date) in Caucasian and Hispanic patients was found to be 3.6 and 5.3 percent (Fernández et al., 2017).

We observed a significant dependence of the ratio of uncollected electronic prescriptions on the age of a patient—both in the overall MN and in the MN and PMN of our sample. This was in concordance with previous studies (Kardas et al., 2020). The exception is the youngest age group (under 18), if not included the dependence would have been even stronger. This might be explained by the specific nature of this group as its behavior is fully under the control of its guardians (we have no way of determining to which group a particular patient’s guardian belongs).

Other authors observed further PMN dependencies (e.g., level of education of the patient (Harsha et al., 2019), socioeconomic disadvantage (Wamala et al., 2007), or patient-provider race and sex concordance (Adamson et al., 2017)). We did not study these types of dependences as the Czech OEPS does not contain any data enabling us to do so.

Previous studies had identified significant differences in PMN between different medication groups with much higher rates in medication related to asymptomatic conditions, such as antihypertensives (Singer et al., 2022) or lipid modifying agents (Raebel et al., 2012). Our study corroborated this only to a limited extent. Compared to the PMN of our studies sample identified a higher PMN in the Psychoanaleptics group and the Lipid modifying group, but the differences to the average PMN are rather small.

Analyzing individual ATC substances, only in two of them its PMN doubly exceeded the PMN of our sample (diltiazem and quinapril), and in further three substances, the PMN surpassed one and half of the PMN of our sample (acarbose, lacidipine, and enalapril). However, the frequency of appearance of these substances in our sample was inadequately low when compared to other substances. Therefore, we could not conclude that the PMN of any particular ATC substance in the Czech Republic was significantly different from the others.

Due to the large and complex study sample, we were able to observe an interesting relationship between MN and PMN in different medication groups, subgroups, and even particular ATC substances. The PMN was twice as high as MN in Lipid modifying agents and the same trend was seen in Antidiabetics, Antithrombotic agents, and Medication for the cardiovascular system. In some antihypertensives (lacidipine, diltiazem, and quinapril) the PMN was three or four times as high as the corresponding MN of these substances. This showed that the initiation of the above-mentioned medications is an issue and physicians and the whole healthcare system should concentrate more thoroughly on this problem.

Conversely, no difference between the PMN and the MN was observed in the Anti-infectives and Psychoanaleptics groups. This affirmed the hypothesis about a lower willingness to initiate a medication related to asymptomatic conditions. However, we were not able to find any other study comparing the PMN and MN, so this topic should be further researched.

Many studies conducted on PMN were focused on different medications or were conducted on a limited sample of patients. Using the Czech OEPS we were able to analyze almost a complete set of outpatient medications prescribed and collected in the whole country. Therefore, we deem our findings very credible. We have found the OEPS data as a useful tool for pharmacoepidemiology research, again in concordance with the previous authors (Aarnio et al., 2019).

The overall PMN in selected medications in the Czech Republic in 2021 reached 4.56 percent. A significant dependence of the ratio of uncollected electronic prescriptions on the age of the patient was observed with the highest PMN in the age group 18–39 years and the lowest in the age group 75 + years. A higher PMN than the average was identified in the Psychoanaleptics group and the Lipid modifying group. However, no significant PMN difference could have been confirmed in any particular ATC substance. In the Lipid modifying agents group, the PMN was twice as high as the MN, a similar trend was observed in some other medications groups, subgroups, and even a substantial number of particular ATC substances. The Czech OEPS has been proven to be a useful tool for research on medical non-adherence.

PMN studies differ not only in basic definitions (such as what is a “new medication”, what it means “medication not obtained” *etc.*) but also in analyzed datasets that might be entirely different. Even when the OEPS is used as the database for the analysis, it may bring different results as OEPS designs and implementation in every country vary significantly. Nevertheless, if the national OEPS is widely used, basic information about a prescription (issued, collected) is stored and every prescription is attributed to a particular patient, a PMN study should be able to deliver reliable results. Therefore, we see our results as not only useful for other studies, but we would like to encourage others to use their OEPS to conduct similar studies which, we believe, will bring comparable results.

Our study has a few limitations. Firstly, it is still possible in the Czech Republic to prescribe medications using classical paper-based prescriptions and we omitted these in our study. As there were 1.5 million paper-based prescriptions collected in 2021 (as compared to over 68.5 million electronic prescriptions collected in 2021) this should not have influenced the outcome of our study in any significant way. In some cases, the electronic prescriptions stored in the Czech OEPS were not matched to a particular patient due to misspelling of identification data. Such prescriptions were not included in our study, but according to the SIDC, this occurred in less than 2 percent of electronic prescriptions. Hence, our study was not significantly affected by these circumstances. Lastly, a prescription could have been issued not to the patient, but to another person. No data were available about this situation, but from our experience, we did not expect this to be common. Therefore, we did not anticipate that this might have influenced our study.

We found that the PMN in selected medications is 4.56 percent, which follows earlier studies with similar methodologies from other countries. A higher PMN in the Psychoanaleptics and Lipid modifying medication groups than in the whole studied sample (*p* < 0.05) was observed. We discovered several substances with a larger difference between PMN and MN—this was not observed in previously published studies.

## Data Availability

The raw data supporting the conclusion of this article will be made available by the authors, without undue reservation.

## References

[B1] Aarnio E. Huupponen R. Martikainen J. E. Korhonen M. J. (2019). First insight to the Finnish nationwide electronic prescription database as a data source for pharmacoepidemiology research. Res. Soc. Adm. Pharm. 16, 553–559. S1551741119303687. 10.1016/j.sapharm.2019.06.012 31253500

[B2] Adams A. J. Stolpe S. F. (2016). Defining and measuring primary medication nonadherence: Development of a quality measure. J. Manag. Care Spec. Pharm. 22, 516–523. 10.18553/jmcp.2016.22.5.516 27123913 PMC10398291

[B3] Adamson A. S. Glass D. A. Suarez E. A. (2017). Patient-provider race and sex concordance and the risk for medication primary nonadherence. J. Am. Acad. Dermatol 76, 1193–1195. 10.1016/j.jaad.2017.01.039 28522045

[B4] Aznar-Lou I. Fernández A. Gil-Girbau M. Fajó-Pascual M. Moreno-Peral P. Peñarrubia-María M. T. (2017). Initial medication non-adherence: Prevalence and predictive factors in a cohort of 1.6 million primary care patients. Br. J. Clin. Pharmacol. 83, 1328–1340. 10.1111/bcp.13215 28229476 PMC5427227

[B5] Bauer A. M. Schillinger D. Parker M. M. Katon W. Adler N. Adams A. S. (2013). Health literacy and antidepressant medication adherence among adults with diabetes: The diabetes study of northern California (DISTANCE). J. Gen. Intern Med. 28, 1181–1187. 10.1007/s11606-013-2402-8 23512335 PMC3744297

[B6] Brown M. T. Bussell J. K. (2011). Medication adherence: WHO cares? Mayo Clin. Proc. 86, 304–314. 10.4065/mcp.2010.0575 21389250 PMC3068890

[B7] Bruthans J. (2019). The past and current state of the Czech outpatient electronic prescription (eRecept). Int. J. Med. Inf. 123, 49–53. 10.1016/j.ijmedinf.2019.01.003 30654903

[B8] Bruthans J. (2020). The state of national electronic prescription systems in the EU in 2018 with special consideration given to interoperability issues. Int. J. Med. Inf. 141, 104205. 10.1016/j.ijmedinf.2020.104205 32492586

[B9] Cheen M. H. H. Tan Y. Z. Oh L. F. Wee H. L. Thumboo J. (2019). Prevalence of and factors associated with primary medication non‐adherence in chronic disease: A systematic review and meta‐analysis. Int. J. Clin. Pract. 73, e13350. 10.1111/ijcp.13350 30941854

[B10] da Costa F. A. Pedro A. R. Teixeira I. Bragança F. da Silva J. A. Cabrita J. (2015). Primary non-adherence in Portugal: Findings and implications. Int. J. Clin. Pharm. 37, 626–635. 10.1007/s11096-015-0108-1 25832675

[B11] Fernández A. Quan J. Moffet H. Parker M. M. Schillinger D. Karter A. J. (2017). Adherence to newly prescribed diabetes medications among insured latino and white patients with diabetes. JAMA Intern Med. 177, 371–379. 10.1001/jamainternmed.2016.8653 28114642 PMC5814298

[B12] Fischer M. A. Stedman M. R. Lii J. Vogeli C. Shrank W. H. Brookhart M. A. (2010). Primary medication non-adherence: Analysis of 195,930 electronic prescriptions. J. Gen. Intern Med. 25, 284–290. 10.1007/s11606-010-1253-9 20131023 PMC2842539

[B13] Harsha N. Kőrösi L. Pálinkás A. Bíró K. Boruzs K. Ádány R. (2019). Determinants of primary nonadherence to medications prescribed by general practitioners among adults in Hungary: Cross-sectional evaluation of health insurance data. Front. Pharmacol. 10, 1280. 10.3389/fphar.2019.01280 31736757 PMC6836763

[B14] Hempenius M. Rijken S. Groenwold R. H. H. Hek K. de Boer A. Klungel O. H. (2022). Primary nonadherence to drugs prescribed by general practitioners: A Dutch database study. Br. J. Clin. Pharmacol. 89, 268–278. 10.1111/bcp.15472 35896043 PMC10087833

[B15] Kardas P. Cieszyński J. Czech M. Banaś I. Lewek P. (2019). Primary non-adherence to medication and its drivers of in Poland: Findings of the analysis of the e-prescription pilot. Pol. Arch. Intern Med. 130, 8–16. 10.20452/pamw.14994 31559971

[B16] Kardas G. Panek M. Kuna P. Cieszyński J. Kardas P. (2020). Primary non-adherence to antihistamines—conclusions from E-prescription pilot data in Poland. Front. Pharmacol. 11, 783. 10.3389/fphar.2020.00783 32528297 PMC7253696

[B17] Karter A. J. Parker M. M. Moffet H. H. Ahmed A. T. Schmittdiel J. A. Selby J. V. (2009). New prescription medication gaps: A comprehensive measure of adherence to new prescriptions. Health Serv. Res. 44, 1640–1661. 10.1111/j.1475-6773.2009.00989.x 19500161 PMC2754552

[B18] Laius O. Pisarev H. Volmer D. Kõks S. Märtson A. Maasalu K. (2018). Use of a national database as a tool to identify primary medication non-adherence: The Estonian ePrescription system. Res. Soc. Adm. Pharm. 14, 776–783. 10.1016/j.sapharm.2017.10.003 29030133

[B19] Raebel M. A. Ellis J. L. Carroll N. M. Bayliss E. A. McGinnis B. Schroeder E. B. (2012). Characteristics of patients with primary non-adherence to medications for hypertension, diabetes, and lipid disorders. J. Gen. Intern Med. 27, 57–64. 10.1007/s11606-011-1829-z 21879374 PMC3250550

[B20] Raebel M. A. Schmittdiel J. Karter A. J. Konieczny J. L. Steiner J. F. (2013). Standardizing terminology and definitions of medication adherence and persistence in research employing electronic databases. Med. Care 51, S11–S21. 10.1097/MLR.0b013e31829b1d2a 23774515 PMC3727405

[B21] Rodriguez-Bernal C. L. Peiró S. Hurtado I. García-Sempere A. Sanfélix-Gimeno G. (2018). Primary nonadherence to oral anticoagulants in patients with atrial fibrillation: Real-world data from a population-based cohort. J. Manag. Care Spec. Pharm. 24, 440–448. 10.18553/jmcp.2018.24.5.440 29694286 PMC10398152

[B22] Singer A. G. LaBine L. Katz A. Yogendran M. Lix L. (2022). Primary medication nonadherence in a large primary care population: Observational study from Manitoba. Can. Fam. Physician 68, 520–527. 10.46747/cfp.6807520 35831084 PMC9842140

[B23] Vrijens B. De Geest S. Hughes D. A. Przemyslaw K. Demonceau J. Ruppar T. (2012). A new taxonomy for describing and defining adherence to medications: New taxonomy for adherence to medications. Br. J. Clin. Pharmacol. 73, 691–705. 10.1111/j.1365-2125.2012.04167.x 22486599 PMC3403197

[B24] Wamala S. Merlo J. Bostrom G. Hogstedt C. Agren G. (2007). Socioeconomic disadvantage and primary non-adherence with medication in Sweden. Int. J. Qual. Health Care 19, 134–140. 10.1093/intqhc/mzm011 17449480

